# Multivariate stability statistics of genotype by environment interaction on fresh yield of cowpea

**DOI:** 10.1038/s41598-025-18797-y

**Published:** 2025-09-12

**Authors:** Mona M. F. Ghazy, Waleed M. B. Yehia, Essam F. El-Hashash, Safwat H. Hatab, Karima Mohamed El-Absy

**Affiliations:** 1 Forage Crops Research Department, Agriculture Research Center, Field Crops Research Institute, Giza, 12619 Egypt; 2 Cotton Breeding and Genetic Department, Cotton Research Institute, Agriculture Research Center, Giza, 12619 Egypt; 3 Agronomy Department, Faculty of Agriculture, Al-Azhar University, Nasr City, Cairo 11651 Egypt; 4 Biology Department, University College of Tayma, University of Tabuk, P.O. Box 741, Tabuk, Saudi Arabia

**Keywords:** GE interaction, Multivariate stability statistics, AMMI, GGE, Correlation, Cowpea, Genetics, Plant sciences

## Abstract

The current study aims to assess the ten cowpea genotypes that are stable and adaptive under three locations during the growth seasons of 2021, 2022, and 2023 using multivariate stability statistics. The fresh cowpea yield (t/ha) using combined ANOVA and AMMI analysis revealed that the environment is the most important factor and that variations between genotypes, environment, and genotype by environment interaction (GEI) were significant (*p* < 0.01). Eight main component axes (PCs) were obtained from the sum of squares of the GEI component. The mean squares for the first five PCs were significant (*p* < 0.05 or 0.01). The Sids location boosted the fresh cowpea yield (t/ha) of every genotype studied throughout all growing seasons, with Sakha and Ismailia locations coming in second and third, respectively. In terms of fresh cowpea yield, the G3 genotype produced the highest mean response, but with moderate stability in the nine environments conditions. G7 was the most stable genotype, with the least amount of yield variation across nine conditions, and was identified by AMMI-based stability parameters. Additionally, according to AMMI and GGE biplot analysis, G7 and G2 genotypes showed excellent stability. Most pairs of AMMI-based stability metrics under study had significant rank correlation coefficients (*P* < 0.05 or 0.01) in a positive direction. Using a GGE biplot polygon of “which-won-where”, the environments were separated into two mega-environments. Using the AMMI model and GGE biplot analysis, the environments E4, E5, and E9 had the longest vectors and the highest fresh cowpea yield (t/ha). To guarantee sustainable progress in cowpea production systems, the study emphasizes the necessity of using a multidimensional strategy for genotype evaluation. This will allow breeders to make well-informed decisions for resilience and productivity under a variety of environmental situations. This raises the prospect of concurrent indirect selection of these traits to take advantage of GEI and increase Egypt’s production of fresh cowpeas.

## Introduction

The cowpea (*Vigna unguiculata* (L.) Walp.) is a self-pollinating, herbaceous, annual grain legume crop that is a member of the Fabaceae family. It is a diploid species with 2n = 2x = 22 chromosomes^1^. Cowpeas are also characterized as a resilient crop since they are widely grown for human consumption and animal feed worldwide, primarily by impoverished farmers in sub-Saharan Africa and the arid and semiarid tropics, where other edible legumes do not flourish^2^. Cowpea is a beneficial staple crop for farmers in tough regions under moisture stress and scorching temperatures since it is drought resistant and adaptable to marginal soil because of its nitrogen-fixing capabilities^3^. Humans eat the grain, tender leaves, and young, fresh pods. Furthermore, the crop’s capacity to fix atmospheric nitrogen makes it perfect for rotational systems, and both fresh and dry leaves are valuable for feeding cattle^4^. Cowpea is an important warm-season legume grown, where 15,417,401 and 1933 hectares were planted in 2023, yielding 634.6 and 3764.1 kg/ha and production of 9784129.38 and 7274.21 tons in the world and Egypt, respectively^5^.

The main objective of plant breeding programs is to increase stability and stabilize agricultural production during conditions (locations and/or years)^6^. The yield performances of genotypes may range significantly depending on the environment in which they are examined^7^. The association between a genotype’s environment and phenotypic expression is known as the genotype-by-environment interaction (GEI). The phenomenon of GEI is a common occurrence in plant breeding projects. Designing the proper breeding protocols for the creation and selection of cultivars that are adapted to target conditions depends heavily on the assessment of GEI^4^. It can be caused by variations in the relative ranking of genotypes/varieties, shifts in the size of genotype-to-environment variances, scale differences between environments, or a combination of these factors^8–10^. According to Fox et al. (1997)^11^, a variety with general or wide adaptation is consistently well ranked over a wide range of conditions, whereas a variety with particular or narrow adaptation is the one whose stability is limited to certain environments. Therefore, the scope of GEI is a significant feature in many breeding projects as it influences not only the choice of areas for selection but also whether variety development should pursue general or particular adaptation^11,12^. The evaluation and understanding of the GEI is a crucial first step in the development of improved plant genotypes^13^. To account for the effects of genetic engineering, breeders evaluate genotypes in a variety of contexts (locations and/or years) to identify those with higher adaptation and high and consistent performance^14^. Multi-environmental trials (MET) are being used to determine which production settings are most suited for particular varieties, in addition to identifying superior varieties that can be recommended to farmers^9,15^. However, it is challenging to comprehend the GEI patterns due to the intricacy of the MET data. Genotypes with less GEI are believed to be stable^16^. Biometricians can use a range of statistical modeling tools, including multivariate stability statistics, to evaluate and comprehend GEI.

Because of their capacity to offer a concise summary, allow for the delineation of a mega-environment, distinguish between different environments, and facilitate the selection of stable and well-adapted genotypes for certain settings, biplot-based models have emerged as the preferred techniques^17^. In both irrigation settings, the genotype (G) main effect plus genotype-by-environment (GE) interaction (GGE) and additive main effect and multiplicative interaction (AMMI) biplot models showed efficacy and similarity in identifying and ranking stable genotypes across environments. In the investigation of the GEI, the AMMI and GGE biplot models functioned well and gave a clear picture of genotype stability behavior in the studied environmental settings^18^. The GGE biplot is better than the AMMI graph in a number of ways when it comes to genotype evaluation and mega-environment analysis. These include: evaluating the genotype by mean vs. stability view; explaining more G + GE; making it easier to visualize which-won-where patterns (AMMI may be deceptive); having the inner-product property of the biplot; being effective in evaluating test environments by discriminating power vs. representativeness (something that AMMI analysis cannot do); and displaying the relative performance of each genotype in each environment^19^. Furthermore, compared to AMMI models, the GGE biplot makes more sense and is more biologically applicable for practice when it comes to explaining the PC1 score, which indicates the genotypic influence rather than the additive main effect^14^. When compared to other AMMI family models, the GGE biplot is typically always close to the top AMMI analysis in most situations^20^.

The primary objectives of this study were (1) to evaluate the stability ability of ten cowpea genotypes across nine environmental conditions (three locations and three seasons) using multivariate stability statistics to identify genotypes with high cowpea yield and the most stable performance for breeding programs. (2) to assess the ecosystems’ suitability for cowpea production to increase output. (3) to look at the associations, similarities, and discrepancies between multivariate stability statistics.

## Results and discussion

### Combined ANOVA with AMMI analysis

A significant difference was seen in the impacts of genotype, environments, and genotype by environment interaction (GEI) (*p* < 0.01) on fresh cowpea yield (t/ha), as indicated by the combined ANOVA with AMMI analysis (Table 1). These findings showed that fresh cowpea yield (t/ha) was influenced by the ten cowpea genotypes and the nine environmental conditions (three locations and three growth seasons) under study. These findings agree with those of Kindie et al. (2021)^21^, Kusi et al. (2025)^22^, and Tariku et al. (2018)^23^, who evaluated 25, 23, and 16 cowpea genotypes in six, four, and seven environments, respectively. These findings imply both the appropriate and desired genetic diversity among the many studied genotypes, in addition to the variation between locations and growing seasons under investigation.

With a value of 86.15%, the environment’s sum of squares had the highest component of all the sums of squares. According to the findings, the environment is the primary source of variation since it contributed significantly to the sum of squares for fresh cowpea production, accounting for the main effect. Atakora et al. (2023)^24^, who obtained comparable observations and connected them to the environment and their interaction, bolster this claim. The genotypes and GEI under investigation came in second and third, with values of 6.54% and 4.54%, respectively. Opposite, it was confirmed that GEI was highly significant and had a noteworthy effect on genotypic performance in various contexts since its sum of squares was higher than the genotypes^25^. This suggests that the fresh cowpea yield (t/ha) varied over time due to significant heterogeneity in the genotypes’ responses. The best genotypes under the studied sites in the growing seasons can be chosen with the help of these differences. Genotype, environment, and GEI all had a substantial impact on cowpea dry matter yield^26^. The GEI component’s sum of squares yielded eight principal component axes (PCs). For the first five principal components (PC1–PC5), the mean squares were significant (*p* < 0.05 or 0.01). The first five components contributed 99.08% of the total variation of the total GEI. The PC1 and PC2 of the interaction captured 40.02% and 23.61% of the total GEI sum of squares, respectively. The most accurate model may be anticipated using the first two PCs since they provided a clear explanation of the degree of significance in the source of variation^24,27–29^. This outcome is consistent with the recommendation made by Gauch et al. (1996)^30^ that the AMMI model be used to estimate the most accurate model utilizing the first two PCs.

According to Eisemann et al. (1990)^31^, considerable GEI should be utilized rather than disregarded. Exploiting GEI entails analyzing and interpreting genotypic and environmental variations to determine the stability of performance of various genotypes over a range of contexts. As a result, stability could be calculated and the type and extent of GEI could be investigated, something that a typical analysis of variance cannot do^32^. While the GE interaction suggested that some genotypes were uniquely adapted to particular environments, the observation of significant main effects of genotypes and environments demonstrated that some genotypes were stable across settings. As a result, selection efficiency and, to a greater extent, variety recommendations for production are impacted by this variation in performance between settings. The GEI impacts can be lessened by choosing stable genotypes in a variety of settings^4^.

**Table 1:** Combined ANOVA with AMMI analysis for fresh Cowpea yield (t/ha) of ten genotypes evaluated across the locations and growing seasons.

S.O.V.	Df	Sum Sq	Sum Sq (%)	Cumulative (%)	Mean Sq	F value	Pr(> F)
Environment (E)	8	59060.89	86.15		7382.61	325.03	0.00
Replication/E	18	408.85	0.60		22.71	2.47	0.00
Genotype (G)	9	4485.81	6.54		498.42	11.52	0.00
G x E	72	3115.56	4.54		43.27	4.71	0.00
PC1	16	1246.74	40.02	40.02	77.92	8.49	0.00
PC2	14	735.60	23.61	63.63	52.54	5.72	0.00
PC3	12	548.70	17.61	81.24	45.72	4.98	0.00
PC4	10	355.51	11.41	92.65	35.55	3.87	0.00
PC5	8	200.39	6.43	99.08	25.05	2.73	0.01
PC6	6	26.68	0.86	99.94	4.45	0.48	0.82
PC7	4	1.06	0.03	99.97	0.26	0.03	1.00
PC8	2	0.88	0.03	100.00	0.44	0.05	0.95
Residuals	162	1487.49	2.17		9.18		

Statistically significant differences at *p ≤ 0.05 and **p ≤ 0.01.

### Mean performance

The fresh cowpea yield (t/ha) data in Table 2 revealed a substantial variety (*p* < 0.05 or 0.01) in the genotype performances when comparing the average values of genotypes with LSD throughout the studied locations in the 2021, 2022, and 2023 growing seasons. Some of the genotypes showed significant rank variations across the different environments, suggesting that there is a lot of genetic variety that can be used to increase cowpea yield^25^. Our results showed that Sids location increased the fresh cowpea yield (t/ha) of all genotypes examined in all growing seasons, followed by Sakha and Ismailia locations. Compared with the growing seasons, the fresh cowpea yield (t/ha) of most genotypes studied increased in the 2023 growing season at the Ismailia and Sakha locations and in the 2021 growing season at the Sids location. According to these results, certain genotypes showed good behavior, as evidenced by their high ability to generate fresh cowpea yield (t/ha) in various environments, while other genotypes experienced more negative consequences. Due to the positive interaction between the temperature and humidity, which can interact in complex ways, with temperature affecting the rate of water loss and humidity affecting the availability of water for plants. Understanding these relationships is crucial for developing effective forage management strategies, such as choosing appropriate plant species. The grand mean fresh cowpea yield (t/ha) was significantly higher at the Sids location than at the Ismailia and Sakha locations, with values of 30.46% and 4.94%, respectively, throughout all genotypes and all growing seasons. The studied locations differed in seasonal temperatures and relative humidity. The growing seasons during which the experiments were carried out also differed in terms of meteorological data. High temperatures and low relative humidity were noted in the Sids region, followed by the Ismailia region and the Sakha region, according to meteorological data. Seasonal temperature and relative humidity levels influenced the performance of certain genotypes, which only flourish in favorable conditions; as a result, even minor deviations from the prevailing climatic conditions can hurt growth and yield^24^. At the Sids location, the G5 and G3 genotypes in the 2021 and 2022 growing seasons produced a maximum fresh cowpea yield with values of 86.59 and 82.88 t/ha, respectively. While G10 had the minimum fresh cowpea yield (t/ha) at all locations in all growing seasons, and the lowest value was observed at the Ismailia location in the 2021 growing season. This may be due to Ismailia location differing in factors like temperature, rainfall, soil type, and sunlight, which can significantly impact a genotype’s growth, development, and yield. Also, a genotype might perform exceptionally well in one location but poorly in another, highlighting the presence of genotype-by-environment interaction. Thillainathan and Fernandez (2002)^33^ state that consistent outcomes in a variety of environments (locations and/or years) may lead to yield stability. The crossover kind of GEI influence was shown by the different yield ranking of genotypes in different environments^34^. The GEI effect was identified as a crossover type (changes in a genotype’s rank from one environment to another) by the inconsistent yield ranking of genotypes from environment to environment (Crossa 1990)^35^. According to the study’s findings, the genotypes that were evaluated may not be able to adapt to all of the environments that were examined^4,36,37^.

**Table 2:** Fresh Cowpea yield (t/ha) of ten selected genotypes across the studied locations in the growing seasons. The genotypes key names can be found in Table 5.

Genotypes (G)	Ismailia				Sids				Sakha			
	2021 (E1)	2022 (E2)	2023 (E3)	Mean	2021 (E4)	2022 (E5)	2023 (E6)	Mean	2021 (E7)	2022 (E8)	2023 (E9)	Mean
G1	41.78	40.36	45.64	42.60	68.61	63.42	62.71	64.91	61.53	62.68	65.29	63.17
G2	36.30	39.69	42.22	39.40	75.22	77.89	68.30	73.80	64.66	67.48	76.06	69.40
G3	43.19	44.09	48.49	45.25	78.47	82.88	69.84	77.06	63.07	73.93	68.03	68.34
G4	34.34	35.70	38.91	36.31	79.68	66.85	66.93	71.15	63.07	70.57	64.94	66.19
G5	43.24	42.49	47.63	44.46	86.59	77.26	68.90	77.58	62.48	65.81	64.61	64.30
G6	36.56	37.75	41.29	38.54	80.72	67.30	65.68	71.23	62.53	62.99	69.39	64.97
G7	36.91	37.68	41.44	38.68	77.45	75.67	71.53	74.88	60.64	62.91	74.01	65.85
G8	33.55	34.87	38.01	35.48	82.53	72.68	75.49	76.90	60.25	68.90	67.48	65.54
G9	31.02	32.15	35.09	32.75	80.20	68.62	65.12	71.31	59.19	66.34	72.45	65.99
G10	27.68	28.06	30.97	28.90	59.83	56.50	59.38	58.57	51.96	57.18	59.03	56.05
Grand Mean	36.46	37.29	40.97	38.24	76.93	70.91	67.39	71.74	60.94	65.88	68.13	64.98
LSD 0.05	7.53	3.19	5.12		2.98	2.48	3.25		4.49	4.37	2.79	1.36
CV (%)	14.59	6.03	8.82		2.73	2.47	3.41		5.20	4.68	2.89	5.20
G P-value	0.03	0.00	0.00		0.00	0.00	0.00		0.03	0.00	0.00	

### GxE heatmap analysis

**Fig. 1:**
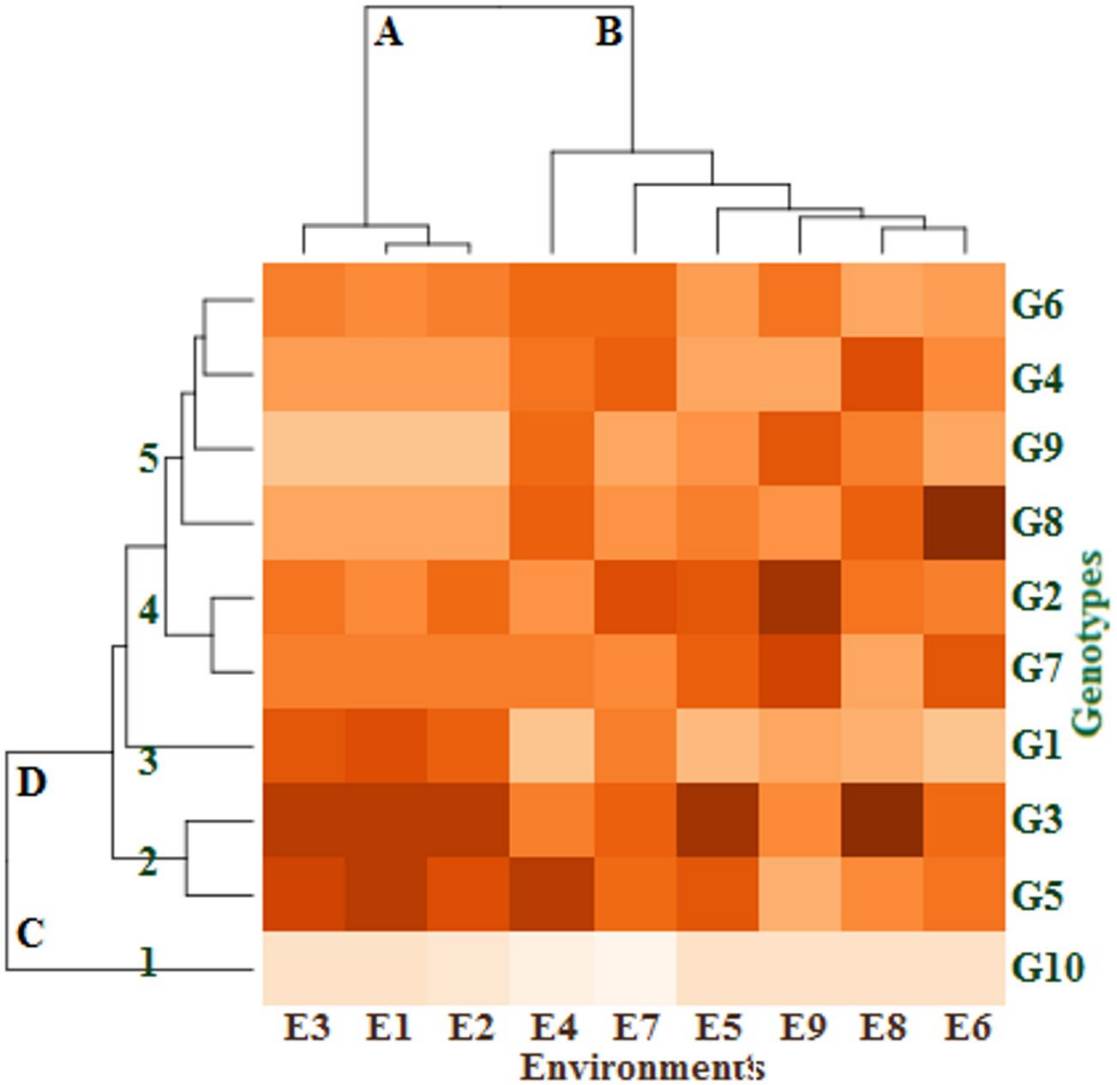
Dendrogram of classified ten genotypes in nine environments by cluster analysis. The color of each block deepens with the increase of the corresponding fresh cowpea yield, then the color depth decreases, and the color scale ranges from mild for the moderate fresh yield to white for the lowest fresh yield. The environments and genotypes key names can be found in Tables 2 and 5, respectively.

As seen in Fig. 1, a visual comparison of the impacts of growing seasons and locations on the genotypes was produced using GxE heatmap analysis of fresh cowpea yield (t/ha). Two dendrograms were identified by the G x E heatmap analysis: the nine environments on top and those that affected the distribution of the ten cowpea genotypes on the left. The environments were divided into two distinct groups using the top dendrogram. E1, E2 and E3 environments made up the group (A) The other environments are included in the group (B) Ten genotypes could be categorized into groups (C and D) based on the left dendrogram. There was only one genotype (G3) in Group (C) Group D was split up into five different clusters. Whereas the genotypes between clusters differ and have the greatest genetic distance, the genotypes within clusters have the least variance and genetic distance. Each of the first and third clusters consisted of one genotype (G10 and G1, respectively). While each of the second and fourth clusters were composed of two genotypes i.e., G3 and G5, as well as G2 and G7, respectively. The fifth cluster consisted of the remaining genotypes (G4, G6, G8 and G9). The G3 genotype gave the highest fresh cowpea yield (t/ha) in the E1, E2, E3, E5, and E8 environments compared with the other studied genotypes in these environments. Also, the genotypes G5 in the E4 environment, G8 in the E6 environment, and G2 in the E9 and E7 environments had the best fresh cowpea yield (t/ha). On the other hand, the genotype G10 had the lowest fresh cowpea yield (t/ha) in all environments under study. Generally, GxE heatmap analysis of fresh cowpea yield (t/ha) classified the genotypes and environments into different separate clusters, and the genotypes G3, G5, G2, and G8 had the best fresh cowpea yield (t/ha) across most environments under study. The findings of the GxE heatmap showed significant variations in fodder yields between genotypes^38^. This suggests that the performance of the genotypes varied among the test conditions. It is possible to speculate that variations in abiotic elements like soil, vegetation, and rainfall could be the cause of the variations in genotype potentials between test environments; climate variations between years could also play a role^22^.

### Multivariate stability parameters

To maximize the benefits of GEI and enhance the accuracy and refinement of genotype selection, yield and performance stability should be taken into account simultaneously^39^. Accordingly, stability statistics can be used as a compromise technique to identify genotypes that are suited to challenging growing conditions and to choose genotypes with a moderate yield and good stability according to El-Hashash and Agwa (2018)^40^. Using a variety of multivariate stability parameters, stability assessments of fresh yield (t/ha) for ten cowpea genotypes under nine environmental variables (three sites in three growing years) were carried out, as shown in Table 3. The G3 genotype gave the highest mean response for fresh cowpea yield with values of 63.55 (t/ha), followed by G5 (62.11 t/ha), G2 (60.87 t/ha), G7 (59.80 t/ha), and G8 (59.31 t/ha) in the nine different environments. The eight genotypes performed better than the grand mean (58.32 t/ha) when the mean response was used as the first criterion for assessing the genotypes under the studied environments. Generally, the previous genotypes recorded the highest values and indicated the most stable genotypes based on the Yi statistic. While, with fresh cowpea yield values of 47.84 (t/ha) and 56.69 (t/ha), the G10 and G9 genotypes showed the lowest mean response, respectively.

The genotypes would be more stable across settings, according to the minimal values of the following statistics: ASTAB, ASI, ASV, AVAMGE, Da, Dz, EV, FA, MASI, MASV, SIPC, Za, and WAAS. The genotype G7 showed a good balance between stability and yield, and it showed above-moderate performance in fresh cowpea yield performance, with the lowest values of all the evaluated multivariate stability parameters. Additionally, the genotypes G3, G5, G8, and G2 demonstrated stability, exhibiting high and moderate fresh cowpea production performance. In contrast, the genotypes G1, G9, and G10 (low yield) showed the greatest multivariate stability parameter values, indicating that they are unstable. According to Pour-Aboughadareh et al. (2022)^41^, only a small number of AMMI model factors, such as MASV, assisted in identifying stable high-yielding genotypes, even though the bulk of them contain a dynamic concept of stability. Using AMMI-based stability metrics, Goa et al. (2022)^25^ reported similar results in cowpea, John et al. (2025)^42^ in rice and Mengistu and Abu (2023)^43^ in sunflower. They concluded that the majority of AMMI statistics are appropriate for identifying stable genotypes. According to Mbeyagala et al. (2021)^44^, our findings thus validate the applicability of ASV in identifying stable and high-yielding cowpea genotypes. ASV is advised for GxE analysis and cultivar identification in cereals and legumes due to its predictive value^45^.

The multivariate stability parameters were used to rank ten genotypes. The results showed that the ranks of genotypes for ASI and ASV parameters, Da and FA parameters, Dz and EV parameters, and Za and WAAS parameters were identical in fresh cowpea yield (Table 3). As a result, it is sufficient to use one of these parameters. Because of this, it might be regarded as a suitable substitute for one another. Additionally, the majority of the multivariate stability parameters under investigation showed frequently comparable ranks for the genotypes, indicating that these parameters are equal for genotype selection. They might therefore be regarded as suitable substitutes for one another. All of the stability measures nearly showed a similar tendency in identifying stable genotypes, according to studies by Anuradha et al. (2022)^46^ in finger millet and Cheloei et al. (2020)^47^ in rice that used the same set of stability indices.

**Table 3:** Multivariate stability parameters and their ranks for fresh yield (t/ha) of ten cowpea genotypes evaluated under nine environments.

Genotype	Y_i_	ASTAB	ASI	ASV	AVAMGE	Da	Dz	EV	FA	MASI	MASV	SIPC	Za	WAAS
G1	56.89	10.63	1.22	5.16	39.28	14.38	0.75	0.11	206.73	1.23	5.43	5.18	0.37	1.61
G2	60.87	5.39	0.52	2.22	21.11	8.78	0.64	0.08	77.12	0.55	3.45	4.89	0.25	1.01
G3	63.55	5.70	0.35	1.48	20.12	8.83	0.66	0.09	77.89	0.46	3.40	4.50	0.22	0.83
G4	57.89	8.85	0.52	2.20	29.11	11.05	0.82	0.14	122.19	0.64	4.62	6.41	0.33	1.28
G5	62.11	5.53	0.26	1.10	20.86	8.33	0.68	0.09	69.35	0.42	3.64	4.77	0.22	0.83
G6	58.25	9.15	0.69	2.92	31.54	11.78	0.79	0.12	138.67	0.70	4.29	4.72	0.26	1.04
G7	59.80	3.59	0.04	0.16	15.84	6.52	0.55	0.06	42.50	0.27	3.19	3.01	0.12	0.44
G8	59.31	5.28	0.30	1.27	20.60	8.02	0.68	0.09	64.28	0.41	3.36	4.94	0.23	0.86
G9	56.69	8.14	0.83	3.52	29.14	11.15	0.79	0.13	124.36	0.85	4.35	5.59	0.31	1.26
G10	47.84	6.37	0.81	3.44	25.90	10.29	0.66	0.09	105.90	0.82	3.88	4.87	0.29	1.19
	Stability parameters rank													
G1	8	10	10	10	10	10	7	7	10	10	10	8	10	10
G2	3	3	6	6	5	4	2	2	4	5	4	6	5	5
G3	1	5	4	4	2	5	3	3	5	4	3	2	2	2
G4	7	8	5	5	7	7	10	10	7	6	9	10	9	9
G5	2	4	2	2	4	3	5	5	3	3	5	4	3	3
G6	6	9	7	7	9	9	8	8	9	7	7	3	6	6
G7	4	1	1	1	1	1	1	1	1	1	1	1	1	1
G8	5	2	3	3	3	2	6	6	2	2	2	7	4	4
G9	9	7	9	9	8	8	9	9	8	9	8	9	8	8
G10	10	6	8	8	6	6	4	4	6	8	6	5	7	7

*Y*_*i*_: mean response; ASTAB: AMMI based stability parameter; ASI: AMMI stability; ASV: AMMI-stability value; AVAMGE: sum across environments of absolute value of GEI modelled; da: annicchiarico’s D parameter; dz: zhang’s D parameter; EV: sums of the averages of the squared eigenvector values; FA: stability measure based on fitted AMMI model; MASI: modified AMMI stability index; MASV: modified AMMI stability value; SIPC: sums of the absolute value of the IPC scores; za: absolute value of the relative contribution of IPCs to the interaction; WAAS: weighted average of absolute scores. The genotypes key names can be found in Table 5.

### Rank correlation among mean yield and multivariate stability parameters

The Spearman’s rank correlation coefficients, which were calculated for each pair of multivariate stability parameters for ten genotypes under nine environments, are shown in Table 4. rASI, and rASV parameters, rDa and rFA parameters, rDz and rEV parameters, and rZa and rWAAS parameters were found to have a highly significant and perfect rank correlation coefficient (*r* = 1.00). According to these findings, the genotype ranks by these stability metrics were the same, as Table 5 illustrates. Mean response showed significant and positive correlations with rASI, rASV, rAVAMGE, rMASI, rMASV (0.05), rZa, and rWAAS (0.01), and these measurements can be utilized to choose high fresh yield and stable genotypes. Also, positive rank correlation coefficients for rYi with the other multivariate stability parameters under investigation were noted. The non-significant relationship between mean yield and stability factors suggests that stability parameters provide information that average yield cannot^48^.

Significant rank correlation coefficients (*P* < 0.05 or 0.01) in a positive direction were found for most pair of multivariate stability measures under investigation. It is confirmed by Anuradha et al. (2022)^46^, John et al. (2025)^42^ and Mengistu and Abu (2023)^43^ that all of the AMMI-based stability parameters showed a positive and significant association with one another. The strong association between these stability statistics is indicated by their significant positive correlation, which also implies that these parameters would have comparable functions in the effective stability ranking of genotypes and vice versa. As a result, these stability models imply that they offer similar information about genotype rankings for stability across different settings. Additionally, stable and high-yielding genotypes can be selected using any one of these factors, or any combination of them. It is not appropriate to interpret these characteristics as distinct processes^49^.

**Table 4:** Rank correlation among fresh cowpea yield and multivariate stability parameters for ten genotypes across nine environments.

Parameters	rY_i_	rASTAB	rASI	rASV	rAVAMGE	rDa	rDz	rEV	rFA	rMASI	rMASV	rSIPC	rZa
rASTAB	0.56												
rASI	0.73*	0.78**											
rASV	0.73*	0.78**	1.00**										
rAVAMGE	0.68*	0.90**	0.87**	0.87**									
rDa	0.60	0.98**	0.88**	0.88**	0.93**								
rDz	0.55	0.73*	0.48	0.48	0.73*	0.68*							
rEV	0.55	0.73*	0.48	0.48	0.73*	0.68*	1.00**						
rFA	0.60	0.98**	0.88**	0.88**	0.93**	1.00**	0.68*	0.68*					
rMASI	0.75*	0.87**	0.98**	0.98**	0.90**	0.93**	0.56	0.56	0.93**				
rMASV	0.65*	0.93**	0.78**	0.78**	0.93**	0.90**	0.78**	0.78**	0.90**	0.88**			
rSIPC	0.58	0.44	0.53	0.53	0.59	0.45	0.72*	0.72*	0.45	0.54	0.67*		
rZa	0.79**	0.82**	0.84**	0.84**	0.90**	0.83**	0.75*	0.75*	0.83**	0.88**	0.93**	0.82**	
rWAAS	0.79**	0.82**	0.84**	0.84**	0.90**	0.83**	0.75*	0.75*	0.83**	0.88**	0.93**	0.82**	1.00**

Statistically significant differences at *p ≤ 0.05 and **p ≤ 0.01; ns: indicate the non-significant difference.

### AMMI biplot

**Fig. 2:**
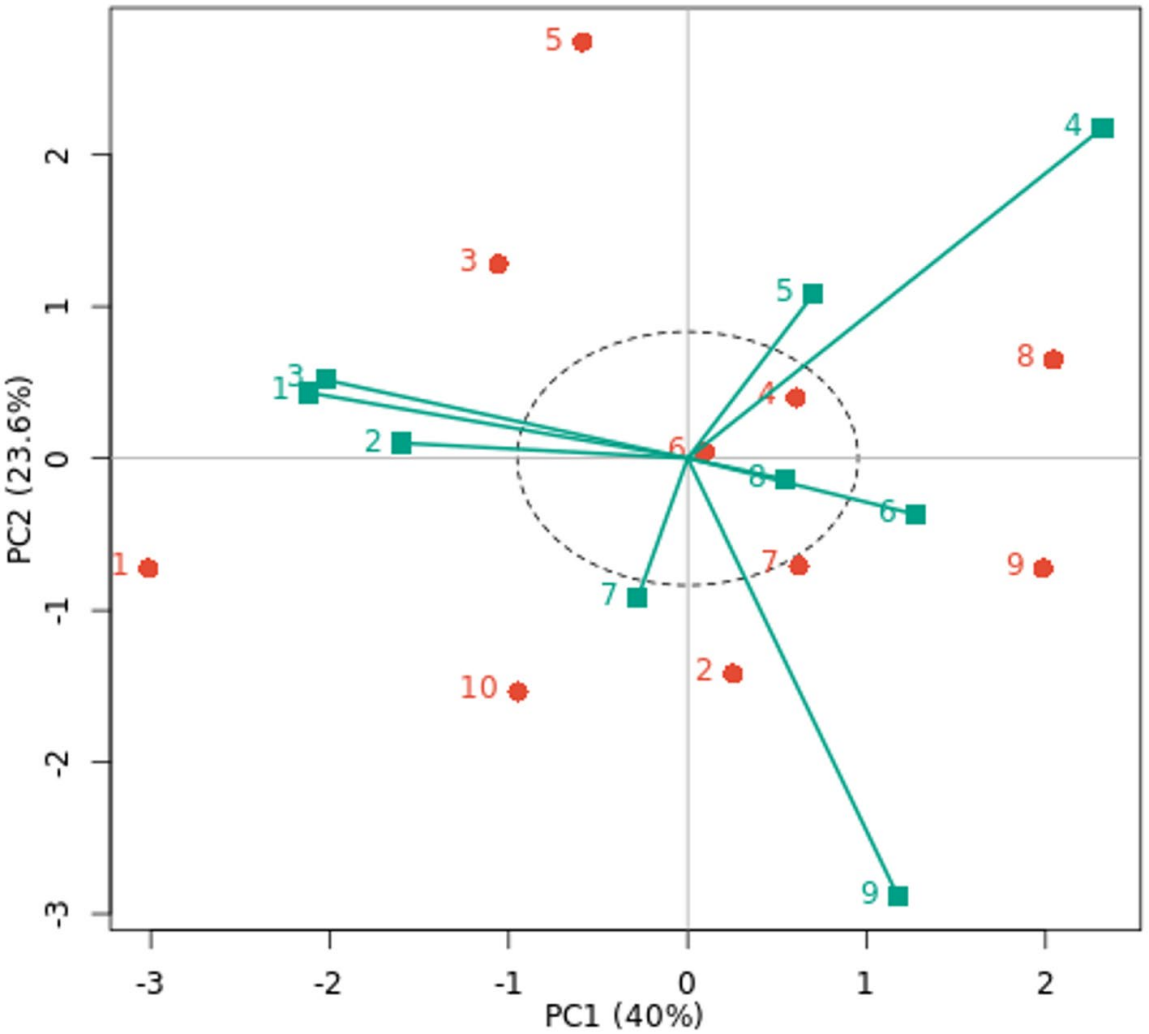
AMMI biplot of PC1 vs. PC2 for fresh yield (t/ha) with ten cowpea genotypes (red color) and nine environments (green color). The environments and genotypes key names can be found in Tables 2 and 5, respectively.

The AMMI biplot was used to analyze fresh yield (t/ha) for ten cowpea genotypes across nine environments, as seen in Fig. 2. Of the ten genotypes under nine environments, PC1 and PC2 explained 40.0% and 23.6% of the total variance, respectively, accounting for the first two PCs’ 63.6% contribution to the entire variability in the data. This suggests that the first two PCs of genotypes and environments predicted the interaction of ten cowpea genotypes with nine environments. Therefore, the best-performing genotypes across nine environments can be found using PC1 and PC2. This is consistent with the advice of Abiriga et al. (2020)^29^ and Zobel et al. (1988)^50^, who suggested that the first two PCs could predict the most accurate model for AMMI. According to Kusi et al. (2025)^22^, the interaction’s PC1 and PC2 accounted for 51.35% and 32.23% of the GEI sum of squares, respectively. In a study by Tunc et al. (2025)^51^, 84% of the variation was explained by PC1 and PC2. This demonstrated that the AMMI model well captured how genotypes performed when exposed to environmental influences. Also, according to Gauch et al. (2008)^17^, cross-validation of the yield variation explained by GEI is possible using the AMMI model with the first and second multiplicative terms. The most stable genotypes that contributed less to GEI were those that were collected and placed near the biplot origin. The genotypes G7, G6, and G4 are stable in all studied environments because they are near the biplot origin with reduced GEI. However, genotypes G10 and G1 are unstable because they are further distant from the origin and contribute more to GEI in all studied environments. The AMMI stability values offer further insight into genotype variation, while the AMMI biplot shows the link between genotypes and environments^27^. Characterizing GEI across cowpea genotypes and identifying genotypes with a favorable balance of stability and improved yield performance were made possible by the application of AMMI^44^.

There is a positive correlation between environment and genotype when they are positioned close to one another in the biplot, suggesting specific adaptation^52^. For example, specific adaptation was demonstrated by the G3 and G5 genotypes with E1, E2, and E5 environments and the G7 and G2 genotypes with the E9 environment. The E4 and E9 environments showed the longest vectors and the highest fresh cowpea yield (t/ha). This implies that the GEI determination is significantly influenced by these environments. Short vectors were seen closer to zero and closer to the biplot origin for the E1, E2, and E3 environments. Additionally, there is less than a 90-degree angle between the E5, E4, and E6 environments and the E7 and E9 environments. Because of this, these environments are less dynamic and more stable, which virtually guarantees that all genotypes will perform better there. These genotypes’ optimal adaptability to their different habitats is demonstrated by the AMMI model’s selection of them in those environments^22^. These results are in line with those for cowpea reported by Ghazy. Mona et al. (2017)^53^, Kindie et al. (2021)^21^ and Kusi et al. (2025)^22^. They saw cowpea genotypes that were wanted in both favorable and unfavorable conditions, as well as genotypes that were solely wanted in either a favorable or unfavorable setting.

### GGE biplot analysis

**Fig. 3:**
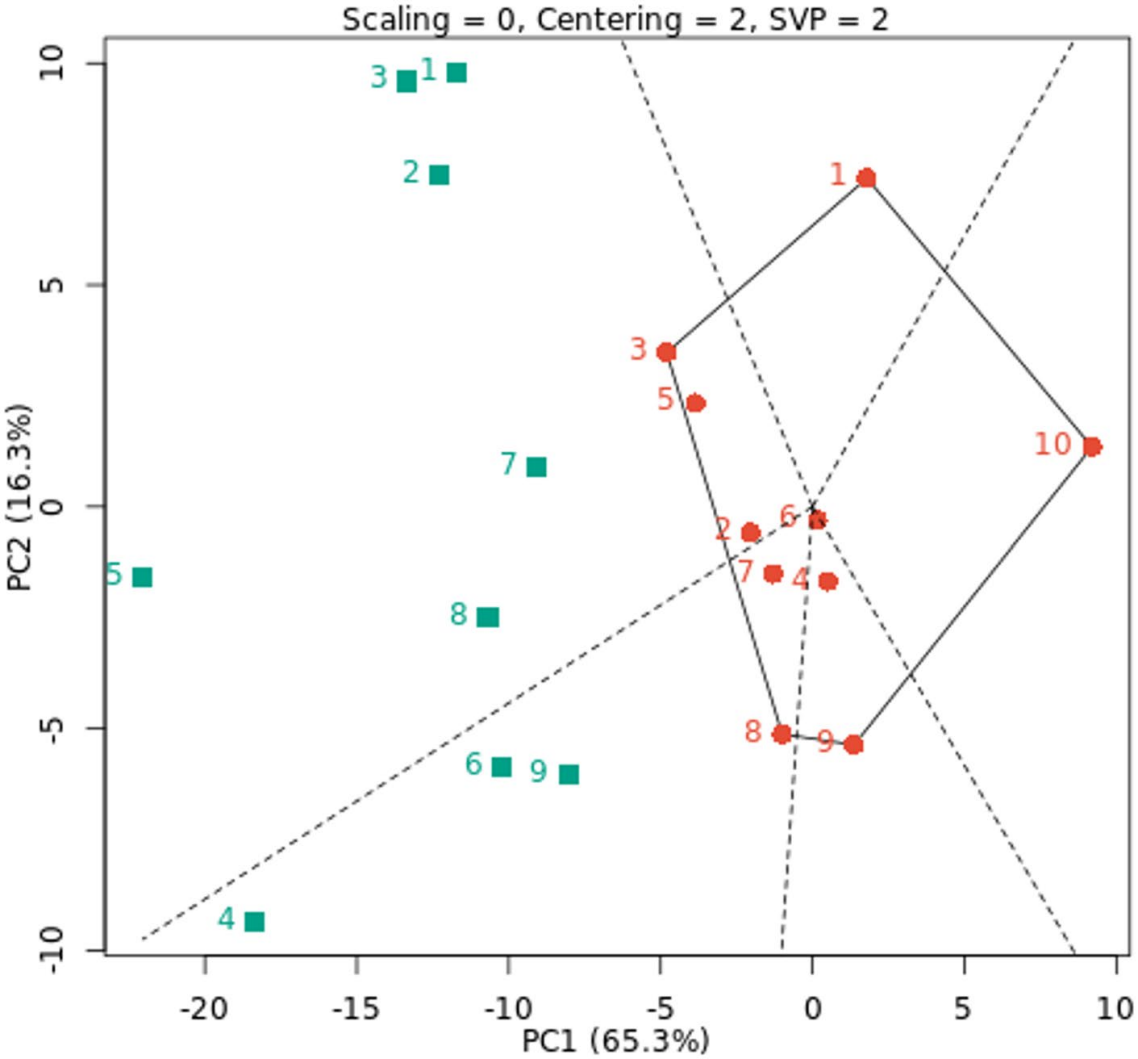
GGE biplot polygon of “which-won-where” for fresh yield with ten cowpea genotypes (red color) and nine environments (green color). The environments and genotypes key names can be found in Tables 2 and 5, respectively.

The GGE biplot was created by plotting the PC1 and PC2, which were produced by applying singular value decomposition to environment-centered yield data^14^. The average environment coordination (AE) technique, which is based on genotype-focused singular value partitioning (SVP), was utilized to assess genotype yield stability using average PCAs in all environments. The GGE biplot is a simple technique for assessing the influence of genotype on the environment in cowpea and offers useful information about the genotypes and environments under study^54^. Both the first two PCs GGE biplots explained 65.3% and 16.3% (81.6%) of the total G + GE variation across the nine environments under study, respectively. According to Kusi et al. (2025)^22^ and Owusu et al. (2020)^55^, the first two principal components accounted for 79.54% and 69.59% of the total yield variance of cowpea throughout the studied settings, respectively, according to the GGE biplot results. Additionally, based on the results by Kusi et al. (2025)^22^, PC1 and PC2 explained 53.68% and 25.86%, respectively. According to Horn et al. (2018)^27^ and Matova and Gasurae (2018)^56^, the GEI can be well explained by the first two major components. In Fig. 3, G1, G3, G8, G9 and G10 genotypes formed the polygon vertices in nine environments under study. These genotypes are dispersed in all directions from the biplot origin, and the polygon encompasses all other genotypes. With the GGE biplot polygon of “which-won-where”, the environments were divided into two sectors, with the top of each sector (vertex) containing the best fresh cowpea yield in the environments located within each sector. Our results were consistent with Yan et al. (2007)^57^, who state that the division of settings into discrete sectors suggests that each sector has its high-yielding genotypes. This implies that the test environments might be combined to form mega-environments, which are larger entities. Using a GGE biplot, the cowpea genotypes were separated into four and three mega-environments, respectively^21,26^. Therefore, the best fresh cowpea yield was obtained by genotype G8 in the E4, E6, and E9 environments and genotype G3 in the other environments. The genotypes inside the polygon are less ecologically fit than those at the vertices. The relations between the different genotypes and the environment was graphically represented by the GGE biplot of the “what won where” analysis. In terms of superior genotypes in connection with surroundings, this observation is consistent with that of Horn et al. (2018)^27^ and Atakora et al. (2023)^24^.

**Fig. 4:**
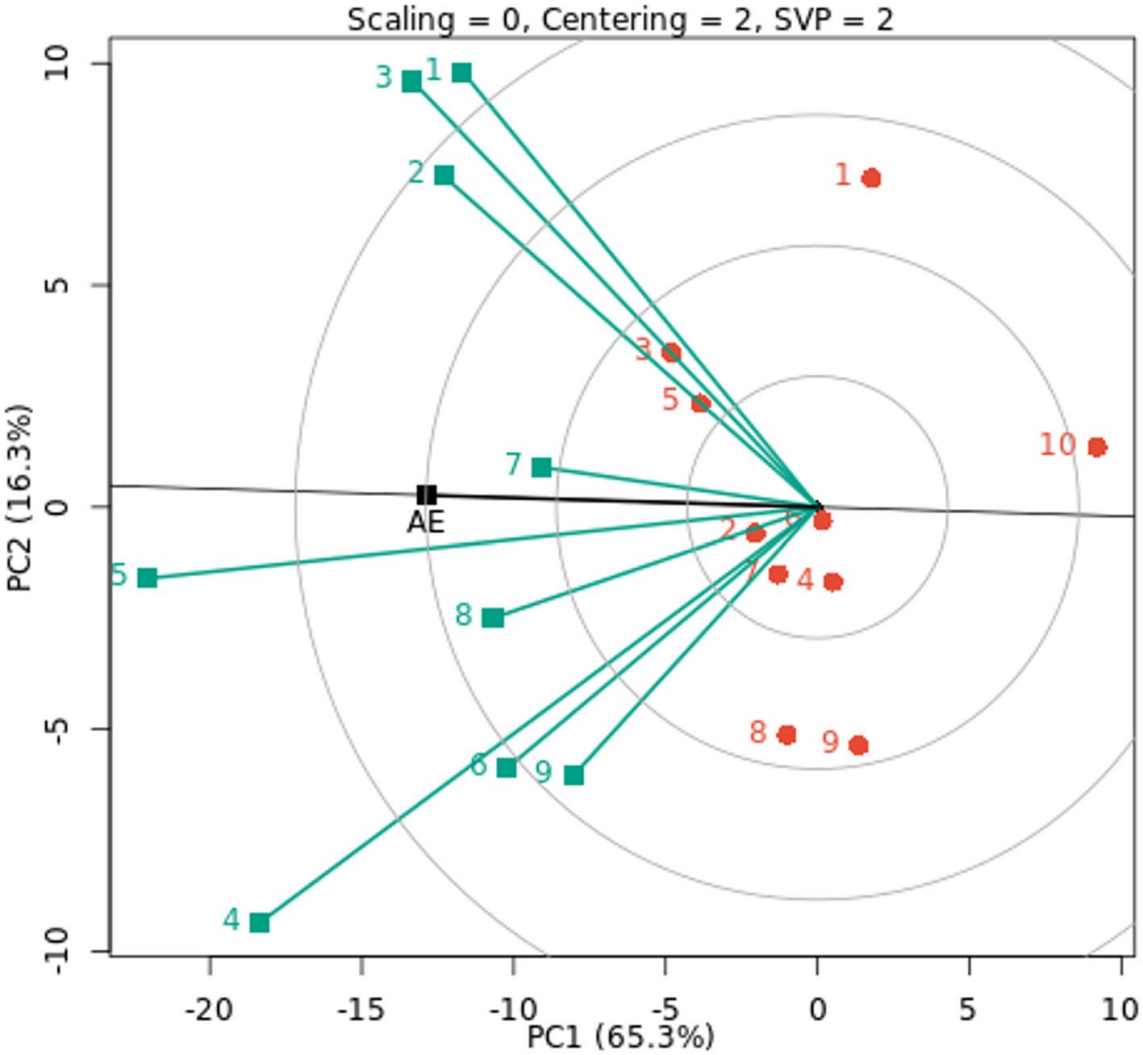
GGE biplot of discrimitiveness vs. representativeness for fresh yield with ten cowpea genotypes (red color) and nine environments (green color). The environments and genotypes key names can be found in Tables 2 and 5, respectively.

The GGE biplot of the decorativeness vs. representativeness view is a useful tool for evaluating test environments and can be used to identify a basic set of representatives and discriminating test environments (Fig. 4). Finding test environments, precisely defining genotype differences, and providing the data required for plant breeders to make an election are all critical. Our findings showed that the test environments E4 and E5 are more representative of other test environments because they have the longest vector and the lowest angles with AE, followed by the environments E1, E2, and E3, as according to Yan et al. (2007)^57^. These findings imply that these environments are optimal and possess the greatest ability to differentiate across genotypes, promoting the selection of superior genotypes for fresh cowpea yield. Additionally, in test environment E7, a tiny angle with AE and a medium vector was discovered. Yan and Rajcan (2002)^58^ state that the most desired genotype is the one that is closest to the graph of the ideal environment. Thus, G7 and G2 are the most stable and productive genotypes in the nine environments under study, as well as genotypes G3 and G5, which are located close to E1, E2, and E3 environments. Strong positive correlations were observed for E7 with E2 and E3, among E1, E2, and E3 environments, and between E5 and E6 environments because the angles between them are acute. E9 with the E1, E2, and E3 environments had a slight positive correlation. While moderate positive correlations were found between the other environments. The cosine of the two environments’ angles and their length vectors indicates how similar (covariant) they are^19^. As a result, the ray lines divided the environments into two groups: the first group included the E1, E2, E3, and E7 environments, while the second group included the other environments under study. According to Yan and Kang (2002)^59^, the optimal genotype is determined by two factors: (i) it has the maximum yield of the complete dataset, and (ii) it is stable because it is found on the AEC abscissa. Nonetheless, the environment is always the primary source of diversity, and plant breeding must give it top priority^60^.

**Fig. 5:**
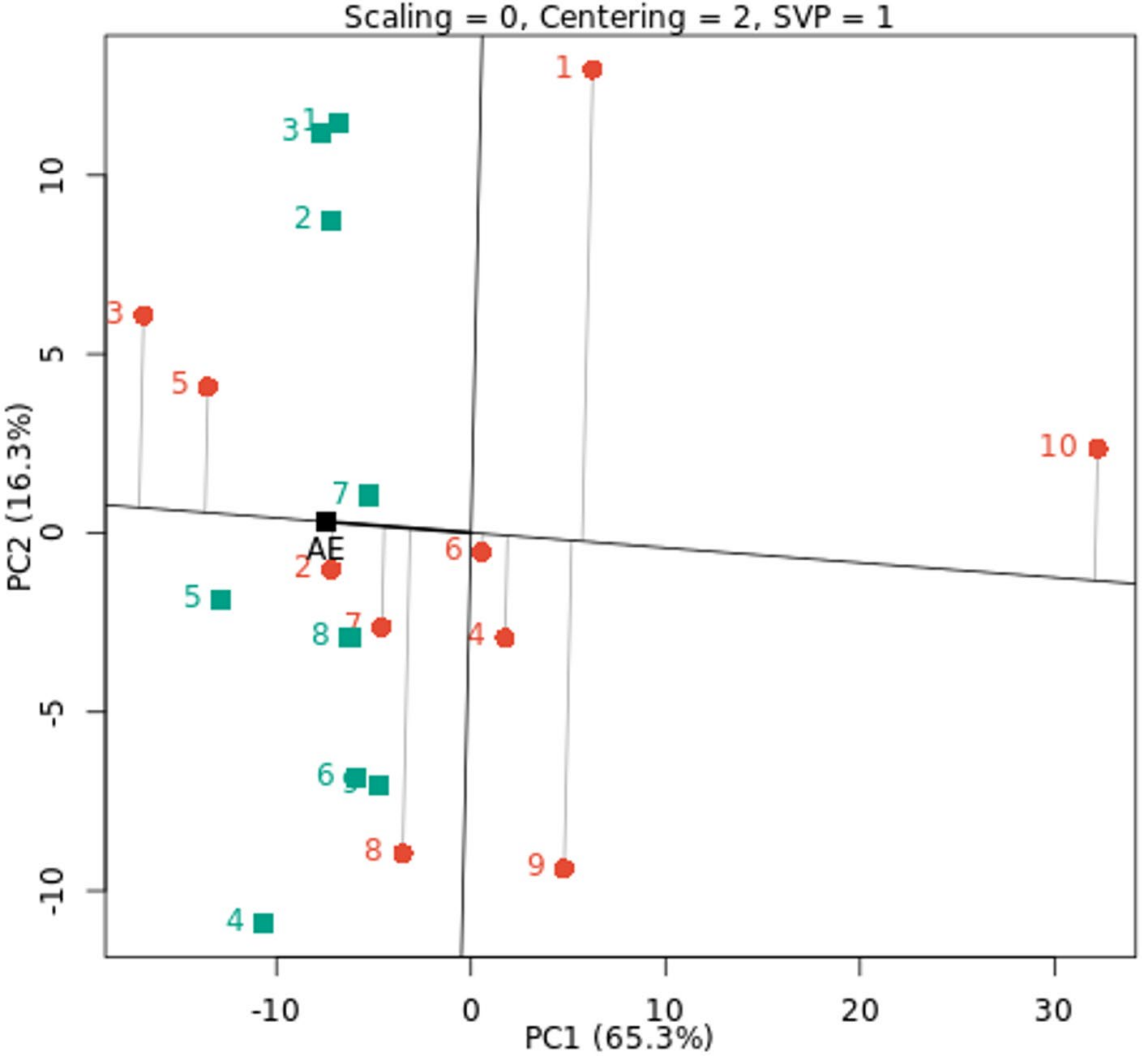
GGE biplot of mean vs. stability for fresh yield (t/ha) with ten cowpea genotypes (red color) and nine environments (green color). The environments and genotypes key names can be found in Tables 2 and 5, respectively.

The representation of genotypes that combine high mean performance and stability is one notable feature of the GGE biplot graph. The genotypes are categorized according to their average fresh cowpea yield, as shown by the arrow sign on the AE (Fig. 5). G6, G4, G1, G9 and G10 genotypes had below-average means, whereas other genotypes had above-average means. Therefore, the lowest fresh cowpea yield was recorded by G10, G9, and G1 genotypes under all nine environments, and are more changeable and unstable. On the other hand, the other studied genotypes were registered as moderate to high for fresh cowpea yield across all nine environments under evaluation. G2 and G7 were the most stable genotypes; they were located almost on the AE abscissa and had a near-zero projection onto the AE ordinate. Compared to the other genotypes, the stable G8, G5, and G3 genotypes yield fresh cowpeas with a moderate to high mean yield. Despite being an effective tool for comprehending GEI, GGE biplot analysis has many drawbacks. This approach disregards environmental impacts even if it is useful for visualizing genotype performance and selecting optimal genotypes and environments^51^.

**Fig. 6:**
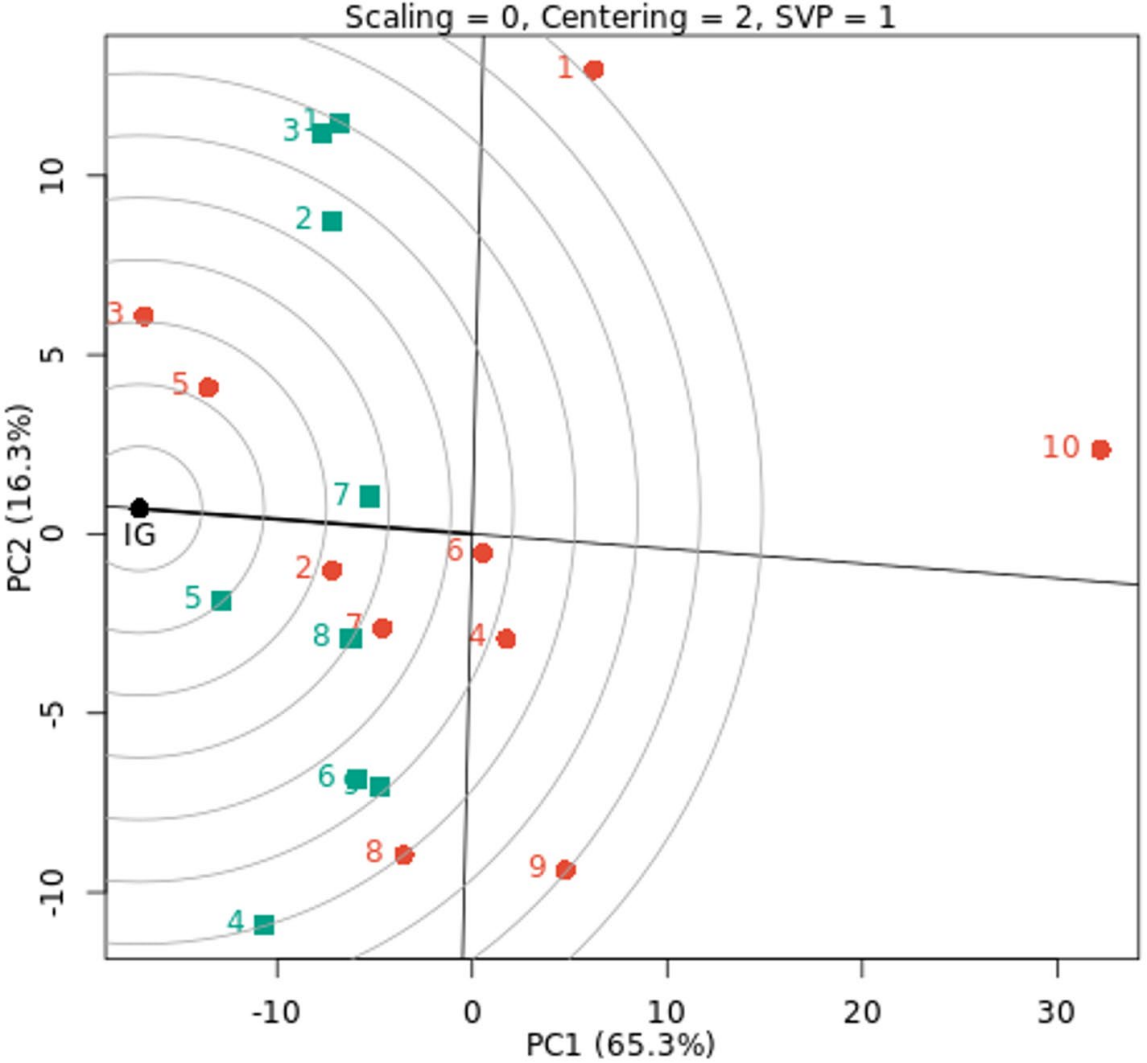
GGE biplot of ranking of the genotypes for fresh yield with ten cowpea genotypes (red color) and nine environments (green color). The environments and genotypes key names can be found in Tables 2 and 5, respectively.

In all environments examined, the genotypes with above-average mean performance were ranked as G3, G5, G2, G7, and G8, as illustrated in Fig. 6. These genotypes are more stable and have higher mean cowpea yields than the others. However, other genotypes, including rank G10, G9, G1, G4 and G6, were unstable and performed below average on average across all situations. Similar findings about the graphical representation of biplots of some genotypes’ performance by comparing various settings have been reported by Horn et al. (2018)^27^ and Atakora et al. (2023)^24^.

Across nine environmental circumstances, the most stable genotypes were G3 by mean response, G7 and G2 by AMMI-based stability parameters, and AMMI and GGE biplot analysis. Because of its capacity to fix atmospheric nitrogen and generate robust roots, the legume family is generally regarded as one of those that can withstand climate change. Regarding G7 and G2, they possess a few key traits that could enable them to adapt to climate change:

1. Hair is present on the leaf’s surface. This makes it more disease-resistant.
2. The thickness of the leaf is considered more than that of other lines.
3. They have the greatest number of branches.
4. The leaf color is darker green than other strains. This indicates that the process of photosynthesis is carried out with higher efficiency.
5. There is a thin, waxy layer on them, which helps prevent quick water loss and is tolerant of drought stress.

## Materials and methods

### Material and experimental procedure

During three successive summer seasons, genotypes were selected from the local cultivar Balady. First, selection was based on the shape and color of grains. Then, fifty genotypes were isolated and labeled from G1 to G50. These genotypes were evaluated for green forage yield, and the best 22 genotypes with the highest green forage yield were identified. In the first season, the 22 selected genotypes were evaluated alongside three control cultivars. In the second season, the top 11 genotypes selected based on their superior yield and its components were further evaluated. Ten cowpea genotypes [*Vigna unguiculata* (L.) Walp.] (Table 5) were selected and evaluated in a randomized complete block design with three replications at the locations in Egypt, i.e. Sakha Agricultural Research Station in Kafr El-Sheik Governorate (Latitude 30°-57’ N and Longitude 31°-07’E), Sids Agricultural Research Station in Beni Sewf Governorate (Latitude 29°3′58.06′′ N and Longitude 31°5′57.79′′ E), and Ismailia Research Station in Ismailia Governorate (Latitude 30° 35’ 30” N and Longitude 32° 14’ 50” E), in the summer seasons 2021, 2022, and 2023. The genotypes consisted of 9 selected lines and the local variety Balady. Genotypes were selected from the local cultivar Balady. First, selection was based on the shape and color of grains. Then we obtained 50 isolated genotypes. They were evaluated on green forage yield, resulting in the best 9 genotypes. Each genotype was sown in a plot with a size of (2.1 × 3) 6.3 m² at the locations in the growing seasons. Each plot consisted of three rows, 3 m long, with 70 cm between rows. The seeds were drilled in rows for each plot at a seeding rate of 35 kg fed^− 1^. Seeds were drilled in May at the locations for the growing seasons. The crop was sown in a single day under uniform field conditions, adhering to all recommended agricultural practices that were applied at the optimum levels for maximum productivity and to minimize environmental fluctuations. Three cuts were taken at 55, 90 and 120 days after sowing at the locations in the growing seasons. The total fresh forage yield in kg plot^− 1^ was calculated by the sum of three cuts, which was subsequently transformed into ton/hectare (t/ha).

**Table 5:** A list of ten Cowpea genotypes investigated across three locations and growing seasons.

No.	Genotypes	Pedigree	Origin
G1	Line1	Selected from Balady	Egypt
G2	Line2	Selected from Balady	Egypt
G3	Line3	Selected from Balady	Egypt
G4	Line4	Selected from Balady	Egypt
G5	Line5	Selected from Balady	Egypt
G6	Line6	Selected from Balady	Egypt
G7	Line7	Selected from Balady	Egypt
G8	Line8	Selected from Balady	Egypt
G9	Line9	Selected from Balady	Egypt
G10	balady	Local variety	Egypt

### Climatic data and soil analysis

Table 6 displays climate data for the growing seasons (May to September) for three planted areas, including minimum, maximum, and average temperature (ºC) as well as relative humidity (%). The climate data during the 2021, 2022 and 2023 growing seasons at the studied locations were provided by the climate change information center and renewable energy, agriculture research center, cairo, egypt. The three growing seasons in the three locations had the lowest and highest minimum, maximum, and average temperature rates over the study period in May and august, respectively. The values of minimum, maximum, and average temperature increased in the 2021 growing season compared with the 2022 and 2023 growing seasons. The highest values of relative humidity were recorded during September (2021, 2022, and 2023) at the Ismalia location; during June (2021), August (2022), and September (2023) at the Sids location; and during August (2021) and May (2022 and 2023) at the Sakha location. While the minimum values of relative humidity were obtained from May (2021 and 2022) and June (2023) at the Ismalia and Sids locations, and from May (2021), September (2022), and June (2023) at the Sakha location. Meteorological data also indicated that the Sids region was characterized by high temperatures and low relative humidity, followed by the Ismailia region and then Sakha region. Table 7 shows the results of soil analysis at three study sites for 0–30 cm depth before planting in three growing seasons (2021, 2022, and 2023) using standard methods by page et al. (1982)^61^.

**Table 6:** Monthly temperature and relative humidity were collected during the 2021, 2022 and 2023 growing seasons at the studied locations, from May to september.

Locations	Months	2021 growing season				2022 growing season				2023 growing season			
		Temperature			RH	Temperature			RH	Temperature			RH
		Min	Max	Mean		Min	Max	Mean		Min	Max	Mean	
Ismaila	May	34.93	17.92	26.42	45.73	31.90	16.55	24.23	48.41	32.03	17.11	24.57	51.80
	Jun	34.62	19.28	26.95	51.07	36.04	20.81	28.43	50.28	35.82	20.77	28.29	49.02
	Jul	38.04	22.42	30.23	48.73	37.09	21.96	29.53	49.76	39.01	22.96	30.99	49.15
	Aug	38.35	23.56	30.95	49.76	37.10	23.41	30.25	53.10	37.67	23.32	30.49	53.90
	Sep	34.56	21.26	27.91	56.64	34.76	21.69	28.23	57.03	36.39	22.66	29.53	53.56
Sids	May	37.53	19.63	28.58	27.27	34.15	17.75	25.95	31.04	34.08	17.97	26.02	33.63
	Jun	36.95	20.51	28.73	33.38	37.40	21.77	29.58	34.36	37.95	22.64	30.30	31.61
	Jul	39.03	23.50	31.27	32.63	37.93	22.27	30.10	33.97	40.25	23.57	31.91	31.68
	Aug	39.37	23.74	31.55	33.12	38.45	23.50	30.98	37.76	39.51	23.75	31.63	36.59
	Sep	35.79	20.89	28.34	43.73	36.86	21.72	29.29	40.80	37.64	22.66	30.15	37.00
Sakha	May	27.68	20.08	23.88	63.37	25.94	18.85	22.40	69.51	33.80	17.19	25.49	50.86
	Jun	28.48	22.11	25.29	65.85	29.41	23.10	26.26	67.43	37.48	20.89	29.18	49.96
	Jul	31.18	25.14	28.16	66.90	30.81	24.61	27.71	66.48	40.95	23.09	32.02	50.19
	Aug	32.45	26.12	29.28	67.30	31.19	25.77	28.48	66.42	39.22	23.45	31.33	53.88
	Sep	30.01	24.75	27.38	64.95	30.45	24.89	27.67	64.72	38.74	23.25	31.00	52.84

**Table 7:** Soil analysis of the experimental fields at Ismaila, Sids, and Sakha locations (0–30 cm depth) before sowing during the 2021, 2022, and 2023 growing seasons.

Locations	Seasons	Mechanical analysis				Chemical analysis				
		Sand %	Silt %	Clay %	Texture	pH	Ec (dS m^− 1^)	N (ppm)	K (ppm)	P (ppm)
Ismaila	2021	73.77	20.00	6.23	Sandy loamy	8.10	0.73	12.00	279	6.81
	2022	68.32	25.83	5.85		9.25	0.16	14.00	264	7.65
	2023	71.05	22.92	6.03		8.62	0.45	11.00	281	6.49
Sids	2021	62.01	20.12	17.87	Loamy sand	8.08	1.52	21.00	16.50	19.00
	2022	59.19	23.84	16.97		8.47	1.07	20.00	17.40	18.00
	2023	60.60	21.98	17.42		8.24	1.29	18.00	16.90	16.00
Sakha	2021	24.00	26.40	49.60	Clay	7.96	1.88	16.00	475	10.00
	2022	26.41	24.38	49.21		8.00	0.54	17.00	399	9.00
	2023	25.21	25.39	49.40		7.95	1.3.0	16.00	465	7.00

Locations	Seasons	Organic matter (%)	Cations meg/L				Anions meg/L			
			Ca^++^	Mg^++^	Na^++^	K^++^	CO_3_^−^	HCO_3_^−^	Cl^−^	SO_4_
Ismaila	2021	0.45	3.50	2.50	1.64	0.50	---	1.00	3.25	3.89
	2022	0.51	2.35	1.98	1.51	0.37	---	1.20	3.10	3.76
	2023	0.48	2.83	2.24	1.53	0.32	---	1.17	3.23	3.80
Sids	2021	1.20	2.73	2.15	13.91	0.38	---	1.76	10.97	6.08
	2022	1.16	1.95	1.49	12.55	0.41	---	2.16	9.98	5.19
	2023	1.19	1.76	1.83	13.44	0.45	---	1.98	10.39	5.69
Sakha	2021	1.55	2.63	1.9	14	0.35	---	1.75	10.48	6.65
	2022	1.50	0.95	0.82	13.72	0.37	---	2.83	9.71	5.80
	2023	1.49	1.73	1.30	13.75	0.40	---	2.40	10.05	5.58

### Statistical analysis

The impacts of genotypes, nine settings (three locations and three seasons), and their interaction on fresh cowpea yield (t/ha) were assessed using the AMMI^62^. F-tests were used to determine statistical tests of significance for these values. Multivariate stability parameters were used to cover a broad spectrum of stability assessment approaches. The multivariate stability parameters were performed in ASTAB: AMMI based stability parameter^63^; ASI: AMMI stability index^64^; ASV: AMMI-stability value^45^; AVAMGE: Sum across environments of absolute value of GEI modelled by AMMI^65^; Da: Annicchiarico’s D parameter^66^; Dz: Zhang’s D Parameter^67^; EV: Sums of the averages of the squared eigenvector values^68^; FA: Stability measure based on fitted AMMI model^69^; MASI: Modified AMMI stability index^70^; MASV: Modified AMMI stability value^71^; SIPC: Sums of the absolute value of the IPC scores^72^; Za: Absolute value of the relative contribution of IPCs to the interaction^65^; WAAS: Weighted average of absolute scores^73^. Additionally, once the significance of the GEI was established, adaptability and phenotypic stability analyses were graphically performed using the AMMI^50^ and GGE biplot^14^ models. Principal component analysis and Spearman’s rank correlation coefficient were used to better comprehend the association between the multivariate stability parameters and all possible pairwise comparisons of fresh cowpea yield (t/ha). The statistical analysis was conducted using PBSTAT-GE 3.0.3^74^.

## Conclusion

In this study, the environment and its interaction with the genetic structure of ten cowpea genotypes substantially impacted the phenotypic expression of fresh yield. Consequently, based on nine environmental conditions, the genotypes have shown significant and notable reactions to fresh cowpea yield. G3 by mean response and G7 and G2 by AMMI-based stability parameters, AMMI and GGE biplot analysis were the most stable genotypes across nine environments conditions. Using the AMMI model and GGE biplot analysis, E4, E5, and E9 are determined to be the best environment for genotype screening. The AMMI model, GGE biplot analysis, and other multivariate stability parameters can offer important insights into the performance of genotypes and help identify genotypes as having strong potential for high fresh cowpea yield across the nine environmental conditions. These genotypes can be recommended as the most stable genotypes with the highest and moderate cowpea productivity under the Sids location. Therefore, these genotypes are utilized in future cowpea breeding programs to develop improved varieties that address conditions in Egypt.

## Data Availability

The data are presented within the manuscript as tables and figures.

## Declarations

**Correspondence** and requests for materials should be addressed to E. F. El-Hashash.
